# A “Pedi” Cures All: Toenail Trimming and the Treatment of Ulcerative Dermatitis in Mice

**DOI:** 10.1371/journal.pone.0144871

**Published:** 2016-01-06

**Authors:** Sean C. Adams, Joseph P. Garner, Stephen A. Felt, Jerome T. Geronimo, David K. Chu

**Affiliations:** 1 Department of Comparative Medicine, Stanford University, Stanford, California, United States of America; 2 (By Courtesy) Department of Psychiatry and Behavioral Sciences, Stanford University, Stanford, California, United States of America; University of British Columbia, CANADA

## Abstract

Ulcerative Dermatitis (UD) is the most common cause of unplanned euthanasia in mice used in research, with prevalence rates reported between 4 and 21%. UD is characterized by a deep, ulcerative lesion that appears most commonly over the dorsal neck and is attendant with an intense pruritus. The underlying cause of UD is currently unknown, and as a consequence, there are no directed therapies that resolve lesions reliably. However, there is a growing body of evidence that suggests a behavioral component to the onset, maintenance, and progression of UD lesions. Scratching behavior in response to the intense pruritus associated with UD lesions may be an effective target for interventional therapies. We hypothesized that interfering with scratching behavior by trimming the toenails of mice with UD, would resolve UD lesions. To test this hypothesis, we first evaluated the efficacy of toenail trims with a single application of Vetericyn at the time of treatment *versus* our previous standard of care, topical Tresaderm applied daily. We found that toenail trims were significantly more effective at resolving lesions (n = 39 toenail trims, n = 100 Tresaderm, p<0.0001) with 93.3% of animals healing by 14 days (median time to lesion resolution). Furthermore, dorsal neck lesions did not recur by 42 days after a single toenail trim (n = 54); however, flank lesions did not resolve and the outcome of the two lesion distributions following treatment were significantly different (p<0.0001). Finally, we implemented toenail trims at an institutional level and found similar efficacies (approximately 90%) for toenail trims regardless of one-time topical supplement used (triple antibiotic ointment, Tresaderm, and Vetericyn, n = 55, 58, 18, p = 0.63). This is the first report of a highly effective treatment for one of the most serious welfare issues in laboratory mice.

## Introduction

Ulcerative Dermatitis (UD) of mice is a pervasive condition commonly seen in mouse research facilities. Given the high prevalence, the nature of the wounds, the lack of effective treatment, and the resulting euthanasia rate, UD is one of the most important welfare issues in laboratory mice. UD can represent over half of the dermatitis cases seen [1]; furthermore, the prevalence of UD in research facilities is between 4 and 21 percent, depending on the demographics of the population examined [2, 3]. When taken in the context of total mouse numbers used in biomedical facilities, the number of animals affected by UD is staggering.

Lesions have a distinctive appearance and are characterized by deep, ulcerative wounds that appear with specific body distributions—most commonly the dorsal neck, followed by facial, flank, limb and other locations [1]. These lesions are coincident with an intense pruritus eliciting up to 25 scratching bouts per minute [4]. Mice with UD spend much of their days literally tearing themselves apart in an apparent attempt to relieve a condition that we don’t fully understand. Multiple factors have been identified that may predispose mice to UD including abnormal grooming behavior prior to lesion onset, age and sex [2, 4–7], excessive grooming progressing to oronasal inflammation [6, 8], T-cell deficiencies [9, 10], environmental factors (alterations in humidity, temperature, and diet) [3, 5–7], infectious agents [11], irradiation [12], immune complex vasculitis [2], and perhaps most importantly, genetic predisposition [13, 14]. Mice of the C57BL/6 background are particularly susceptible to the disease and are responsible for the higher end estimate of prevalence reported [4]. Yet, for all of these associations, the trigger that incites lesion appearance is not clearly identified and is likely multifactorial in nature.

A major drawback of our lack of understanding of the proximal cause of UD is the lack of directed therapies for this disease. Current treatment options target symptomatic relief of the clinical signs, chiefly pruritus and ulcerations, and are highly variable in efficacy. Topical and systemic therapies rely on the principles of anti-inflammatory mediated analgesia, alleviating pruritus, anti-oxidative protection, immune suppression, antisepsis, and local anesthesia [3, 15, 16]. Efficacy of these therapies (when defined by total resolution of lesion) varies by study, but never exceeds 65% of affected animals [15]. Besides a lack of efficacy, these therapies frequently require animal care providers to travel to multiple facilities per institution in order to provide daily treatment to each affected animal, representing both a significant burden with regards to manpower needed as well as a major welfare concern as these treatments appear to fail more often than not. At our own institution, when these strategies fail, euthanasia is the only option and results in a significant loss of animal life, research efforts and results, and has negative financial impacts. In particular, scientists working with older animals often plan on the loss of a significant proportion of the animals entering the study resulting in an avoidable increase in animal numbers and considerable expense.

An area of underserved UD research is the assessment of behavior-oriented interventions. Such strategies are reported in laboratory animal science trade magazines and poster presentations but are absent from peer-reviewed publications. These strategies include mechanical interventions for the interruption of scratching behavior by mice with UD. For instance, the use of Stomahesive as an additional skin barrier to prevent the mechanical damage induced by hindlimb toenails during scratching was reported to aid in lesion resolution [17]. In a similar vein, green clay has been used to coat UD lesions and prevent trauma from scratching [18]. Finally, the most straightforward intervention, employed in both veterinary and human medicine alike for patients with intense pruritus, nail trimming, was described as a successful intervention for UD [19]. However, all of these interventions are reported only in conference abstracts, and have not been reported in peer-reviewed journals. Furthermore, these interventions are rarely employed due to concerns regarding practicality [20]. Often, they require significant time to perform (e.g. performed under anesthesia, using multiple animal care providers, or require multiple treatments per day) which limits scalability in a large research setting.

Barbering, a commonly seen behavior in socially housed mice, has been identified as a compulsive behavior, lending credence to the concept of UD having a behavioral component [21]. Furthermore, mice with oronasal inflammation owing to excessive barbering were highly associated with UD lesions providing further support for a behavioral link [8]. The most convincing argument for behavioral involvement lies in the observation that elevated scratching behavior prior to lesion onset is predictive for UD development [6]. Together, these discoveries place behavior as a central feature of UD that may be intimately tied to the itch-scratch cycle.

The itch-scratch cycle appears to play a large role in elicitation of the behaviors that maintain UD lesions. This cycle describes a circuit by which an itch sensation evokes a scratch response, which in turn, results in the traumatic elaboration of pro-inflammatory mediators, which serve to reinitiate the cycle again [22]. Maropitant citrate, which blocks the binding of substance P to the tachykinin neurokinin 1 (NK1) receptor which is involved in itch sensation results in diminished pruritus, limited UD lesion resolution, and may partially inhibit the itch-scratch cycle [20, 23, 24]. While many of the UD therapies mentioned above may in one capacity or another interfere with the itch-scratch cycle, the penetrance into that cycle appears to be incomplete as relapse and progression of lesions are a common outcome. The more promising behavioral intervention strategies reduce the inflammation associated itch-scratch cycle by reducing the potential for trauma from ongoing scratching behavior, which may result in a more robust recovery. Hence our interest in examining toenail trims as a treatment for UD.

We have hypothesized that toenail trims, as a mechanical intervention in the itch-scratch cycle, will interfere with the maintenance and progression of UD lesions. In order to test this hypothesis, this study investigates the short and long term efficacy of toenail trims on UD lesion resolution. Our first aim towards efficacy assessment was to compare toenail trims with our institute’s current standard of care, the daily administration of a topical anti-inflammatory. We expected that removing the sharp, dagger-like tips of pelvic limb toenails would interfere with the itch-scratch cycle providing adequate time for ulcerative lesions to heal. The second aim assessed whether lesions reappeared following a single toenail trim and subsequent toenail regrowth. We expected that lesions would eventually reappear after a toenail trim given that the treatment does not mitigate any other processes occurring in UD other than the trauma of scratching itself. Finally, we sought to refine the technique in order to make implementation at an institutional level practical and feasible. This required devising a strategy that was effective, fast, simple, and economical. We hypothesized that toenail trims could safely and quickly be performed on conscious mice using a restraint device that immobilized the targeted digits by fully extending the pelvic limbs. This method would avoid the need for anesthesia, which is time- and resource- limited in a research setting, and could be performed by a single person without causing undue stress to the mouse. Here we present findings that have a profound impact on mouse welfare with regards to a disease that causes untold distress, is the leading cause of death in aged mice [2], and impacts hundreds of thousands of animals each year.

## Materials and Methods

### Ethics Statement

The study protocol was approved by the Stanford University Administrative Panel on Laboratory Animal Care (IACUC) and was performed in accordance with *Guide for the Care and Use of Laboratory Animals* (8th edition). All animals in this study were previously enrolled in other IACUC-approved protocols and this study was performed on those animals identified as clinical cases by the Veterinary Services Center. When the veterinarian determined need and cause for humane euthanasia, mice were euthanized by carbon dioxide inhalation. Criteria for early euthanasia included comorbidities that met endpoint criteria of concurrent studies (these animals were excluded from our nail trim study) as well as UD specific criteria, which included the following: lesions greater than 2 cm^2^, moist ulcerative lesions affecting the ventral neck or periocular regions, and lesions that had contracted causing impaired ambulation.

### Housing, Husbandry, and Health Status

The animals used in this study were concurrently enrolled in a number of animal protocols with a wide variety of experimental conditions, housing, and genetic backgrounds. We did not seek to standardize by strain, location, or experimental variables due to concurrent protocol needs, as removing animals from their current study would interfere with ongoing research. Additionally, by utilizing animals with a wide variety of backgrounds and conditions, the applicability of our treatments would be more robust and translatable to other large biomedical research facilities considering the heterogeneity of the animals assessed [25, 26]. Consequently, animals were housed in either corncob (¼ inch, Harlan Teklad, 7097, Indianapolis, IN) bedded static or individually ventilated cages from two manufacturers (Tecniplast, VA, Italy; Innovive, San Diego, CA). Individually ventilated cages underwent approximately 75 complete air changes per hour. Singly housed animals and mice with UD lesions were provided with environmental enrichment consisting of Nestlets (Ancare; Bellmore, NY), and/or shredded paper, and/or paper tubes. Cage change was performed at least every two weeks and spot changed as necessary. Animals were provided with water prepared by different treatments depending on facility; some animals received reverse osmosis treated water prepared at the facility, while others were provided bottled reverse osmosis treated and acidified water prepared by the vendor (Innovive, San Diego, CA). Standard feed (Harlan Teklad, Indianapolis, IN) was provided *ad libitum*, or replaced with a breeder diet for breeding animals (Harlan Teklad, Indianapolis, IN). Rooms were on a 12:12 hour light/dark cycle schedule, maintained between 70-74F, and kept within 30%-70% humidity.

A bioexclusion program was used to assess mouse pathogen status in each facility. At minimum, following a quarterly testing schedule, sentinel mice exposed to dirty bedding were tested and considered free of Mouse Parvovirus (MPV), Minute Virus of Mice (MVM), Mouse Hepatitis Virus (MHV), Enzootic Diarrhea of Infant Mice (EDIM), Theiler’s Murine Encephalomyelitis Virus (TMEV), mites (all species), lice (all species), and pinworms. Our barrier facility excluded the additional pathogen Mouse Norovirus (MNV) on this testing schedule. Furthermore, the barrier facility tested every six months for the presence of *Helicobacter spp*. and *Pastereulla spp*. which were also excluded pathogens. On an annual schedule, sentinel mice were tested and considered free of Sendai Virus, Mouse Adenoviruses 1 and 2, Ectromelia Virus, Lymphocytic Choriomeningitis Virus (LCMV), Pneumonia Virus of Mice (PVM), Reovirus 3, *Mycoplasma pulmonis*, *Spironucleus muris*, and *Giardia muris*.

### Study Design & Enrollment Criteria

This study was performed across three experiments.

#### Experiment 1

To assess the effectiveness of toenail trimming as a treatment, we performed a records review of a randomly selected subset of all mice treated for UD between November 2013 and 2014. This represents a very diverse population of animals across five facilities during the time in which we were testing toenail trimming. Enrollment of animals into the records review study was based on the following criteria: lesions, as determined by a veterinarian, were consistent with UD appearance and body distribution, ectoparasite status was negative by fur pluck and microscopy, animals had observable pruritus, and animals did not have any other identifiable comorbidities. Mice were essentially randomly allocated to two treatment groups: Tresaderm (active ingredients: 40 mg thiabendazole, 1 mg dexamethasone, and 3.2 mg neomycin) (Merial, Duluth, GA) applied daily (our standard topical treatment at the time); or toenail trim with a single application of Vetericyn (active ingredient: neutral buffered 0.015% hypochlorous acid) (Innovacyn, Rialto, CA) reflecting the preferred treatment of the individual veterinarian assigned to the case. From the case records, we were able to reliably identify the treatment, the date treatment was first applied, the number of days to outcome, and the final outcome (cure or euthanasia). Records for a total of 137 mice were examined, representing 39 toenail trim treated animals and 98 Tresaderm treated animals.

Tresaderm treatment consisted of applying approximately 0.25mL directly on the lesion once a day (excluding weekends) for 7–14 days. Toenail trims were performed by the following method: mice were briefly anesthetized using isoflurane gas anesthetic (Henry Schein, Dublin, OH) and the toenails of digits P2, P3, and P4 of both pelvic limbs were trimmed using Spencer Stitch Removal Scissors (Miltex, Plainsboro, NJ) at the level of the hyponychium (nail quick). Based on previous experience in our group, trimming the nails of P2, P3, and P4 was more efficient and resulted in same impact on lesion resolution as trimming toenails from all five phalanges. Occasionally, the hyponychium was cut resulting in mild hemorrhage that was managed by direct pressure and styptic powder with benzocaine (ARC Laboratories, Atlanta, GA). A single application of Vetericyn was applied to the lesion and the animal was allowed to recover.

#### Experiment 2

As toenails regrow within a few days, we wanted to know if UD lesions recurred over time. Furthermore, in the course of treating animals with toenail trims, we noticed that a small number of animals presented with flank lesions in addition to the more typical head-and-neck lesions. It was our impression that the flank lesions did not resolve. We therefore followed 54 animals from initial treatment (day 0) by toenail trim with single application of Vetericyn, scoring them for the presence of flank and/or head-and-neck lesions every two weeks up to 42 days post treatment. Lesions were documented by briefly anesthetizing mice with isoflurane gas and using a digital camera (Sony HDR-XR260V, Sony, New York City, NY) to capture images for lesion assessment.

#### Experiment 3

We next wanted to confirm that the effect of toenail trim in the record review was not due to the single application of Vetericyn. We also wanted to test the efficacy of toenail trim when performed by five different operators across multiple facilities in our institution. Therefore, following the adoption of toenail trim as our standard treatment for UD, we followed the medical records of animals diagnosed with UD. Technical staff performed all trims, however, we allowed different case veterinarians and technical staff to use their topical treatment of choice (Bacitracin-Neomycin-Polymyxin B with Hydrocortisone [BNP-HC], N = 55; Tresaderm, N = 58; or Vetericyn N = 18) as a one-time adjunct on the first day of treatment.

**Fig 1:**
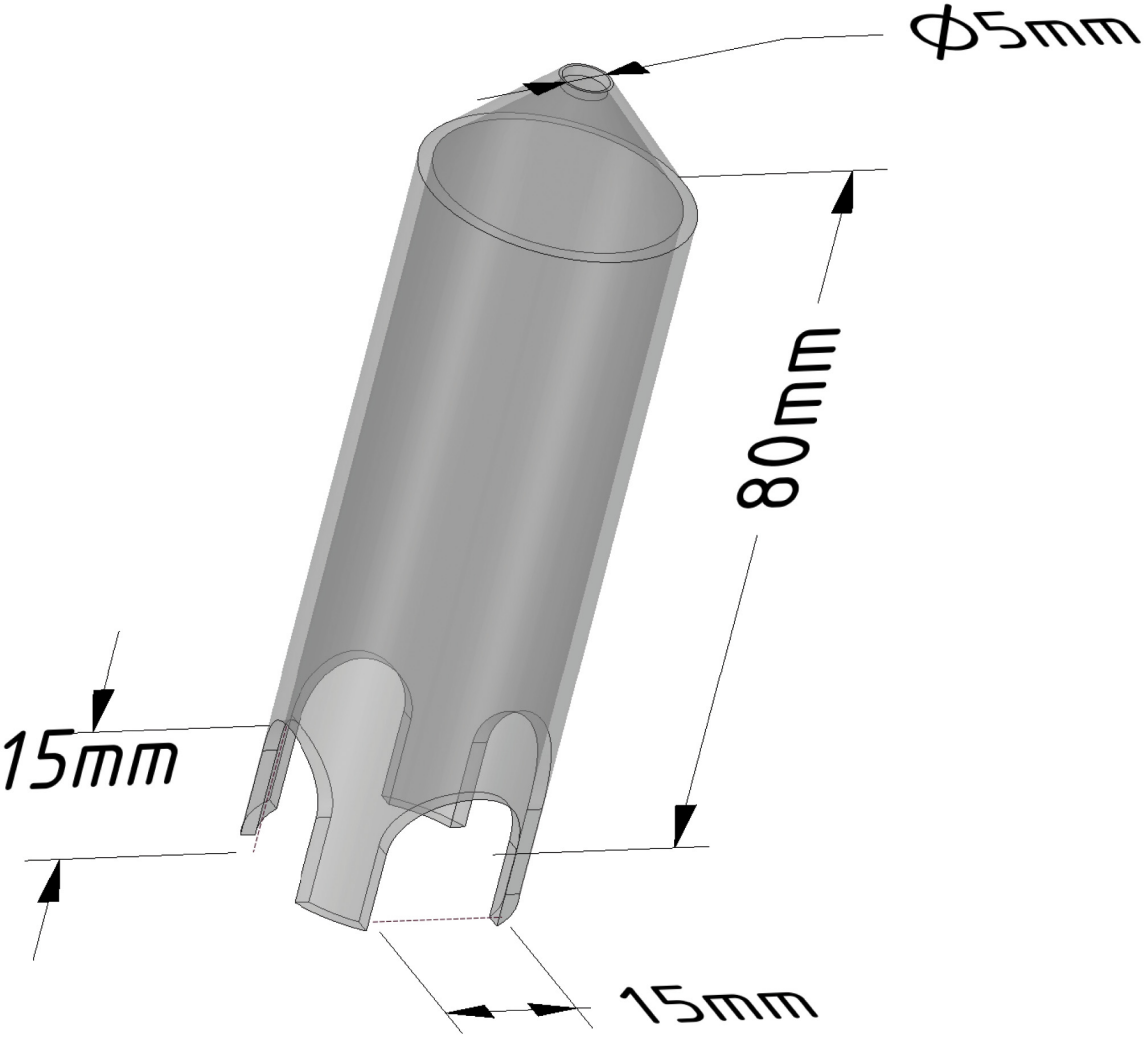
Diagram of restraint device used for toenail trim. A 50mL conical tube was modified by the following changes. Cutouts were made along the rim of the tube to allow seating of the mouse knee against the edge and were lightly melted to remove sharp edges. The tip of the tube was removed and lightly melted to ensure air passage as well as facilitate decontamination between uses. Approximate dimensions are shown in millimeters.

Toenail trims were performed without the use of anesthesia for this experiment and were facilitated by the use of a modified 50mL conical tube (VWR International, Radnor, PA). These custom modified restrainers consist of cutouts to engage the pelvic limb and a port to facilitate disinfection (see Fig 1). Any sharp edges made during the modification were smoothed out by lightly melting the plastic in a fume hood. Mice were gently scruffed and introduced to the restraint device headfirst. The pelvic limbs are manipulated into the cutouts and the knee is placed against the cutout edge. After placement, the extensors of the limb are engaged and the toes easily manipulated for toenail trims.

### Data Processing and Statistical Analysis

Experiment 1: We first tested for the overall effectiveness of the two treatments in terms of lesion resolution (and thus overall survival rates), using a logistic regression implemented with PROC GENMOD in SAS 9.4 for Windows. The analysis tested treatment, stratified (controlled) for the date of treatment (as UD shows season influences), and for the number of days to outcome (to protect against any influence of early euthanasia). We also performed a Kaplan-Meier survivorship analysis on the same data. For this analysis, animals with lesion resolution were right-censored (i.e. removed from the pool of at risk animals). This technique has the advantage of showing the timeline of mortality and cure. However, it also has two distinct disadvantages. First, it is not able to control for confounding factors. Second, it overestimates mortality, particularly when the majority of animals are cured and thus censored (for instance, at the point where all but two animals show lesion resolution, a single mortality event is depicted as 50% of the at risk population dying).

Experiment 2: Using a repeated measures logistic regression implemented as a GEE in PROC GENMOD, we tested whether lesions resolved over time, and whether these curves differed according to lesion type. We initially modeled time as an auto-regressive within-subject categorical effect, to test for recurrence. However, no evidence for recurrence was seen. Therefore, the final analysis treated time as a continuous effect. Post-hoc Bonferroni-corrected planned contrasts were used to investigate significant interactions.

Experiment 3: As with the records review data, we performed a logistic regression implemented in PROC GENMOD, controlling for time of year and duration of treatment, and testing for differential cure rates according to the topical supplement used on the first day of treatment. As before, we also performed a Kaplan-Meier Survivorship analysis, but in this case as we were interested in time to lesion resolution (almost all mice had resolved lesions), cure was treated as the failure event, and euthanized animals were right-censored.

## Results

### Records Review

**Fig 2:**
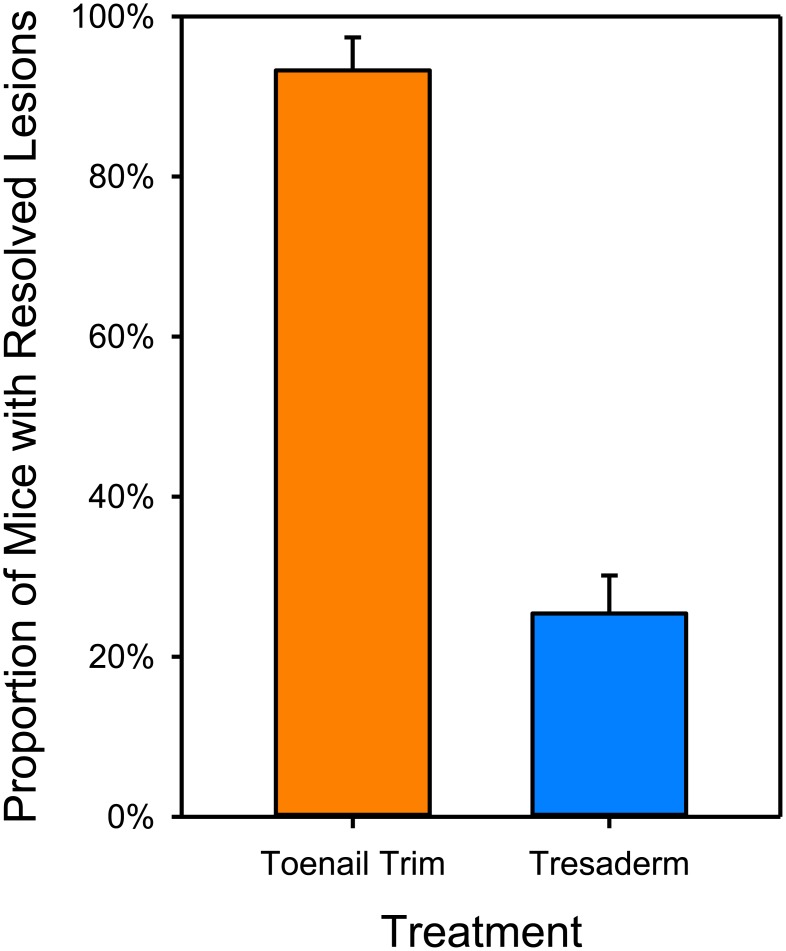
Proportion of mice showing complete lesion resolution by treatment type. Logistic regression analysis showing the proportion of all mice entering treatment that were cured rather than euthanized in the records review. Estimated means and standard errors are shown from the logistic regression (which controls for date of first treatment, and duration of treatment), P < 0.0001.

**Fig 3:**
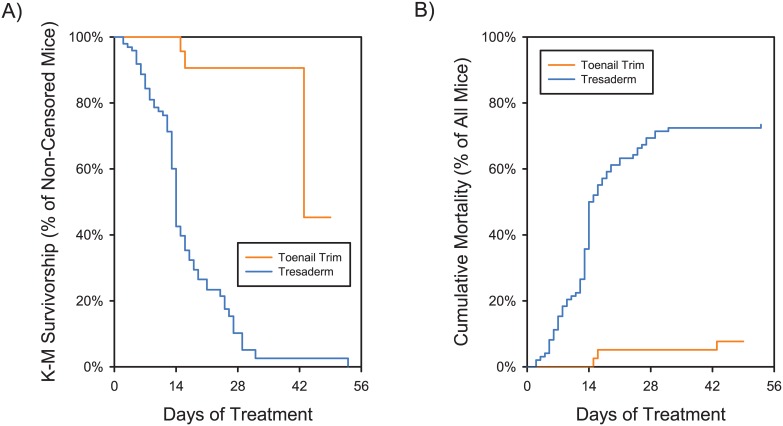
Survivorship and cumulative mortality as a function of time by treatment group. A) Kaplan-Meier survivorship plot. Survivorship is shown as the proportion of non-censored (at risk) animals surviving at each time point (rather than the proportion of all animals surviving). Cured animals are censored (i.e. removed from the total count at each time point), thus the three steps seen in the curve for toenail trim treated animals represent the individual euthanasia events for the three (out of 39) mice which were not cured. The rapid drop in survivorship at day 43 for toenail trim animals reflects the fact that only two animals remained at this timepoint (and the final animal was cured on day 49). B) Cumulative mortality, showing the raw mortality events, without the artifacts injected by Kaplan-Meier survivorship calculations.

Logistic regression showed that the cure rate for mice receiving toenail trim (93.3% +/- 4.1%) was significantly higher than those receiving Tresaderm (25.4% +/- 4.8%) (Likelihood-Ratio ChiSquare = 44.26; P < 0.0001; Fig 2). None of the control variables were significant. The Kaplan-Meier survivorship analysis showed a marked difference in median survival time. For those animals that were not cured, the median time to euthanasia was 14 days for Tresaderm treated mice, and 43 days for toenail trim treated mice (Log-Rank ChiSquare = 40.64; p < 0.0001). Fig 3 shows both the conventional Kaplan-Meier survivorship curves, and the cumulative mortality (to avoid the artifacts of censoring).

### UD Recurrence, Lesion Type, and Treatment Efficacy

**Fig 4:**
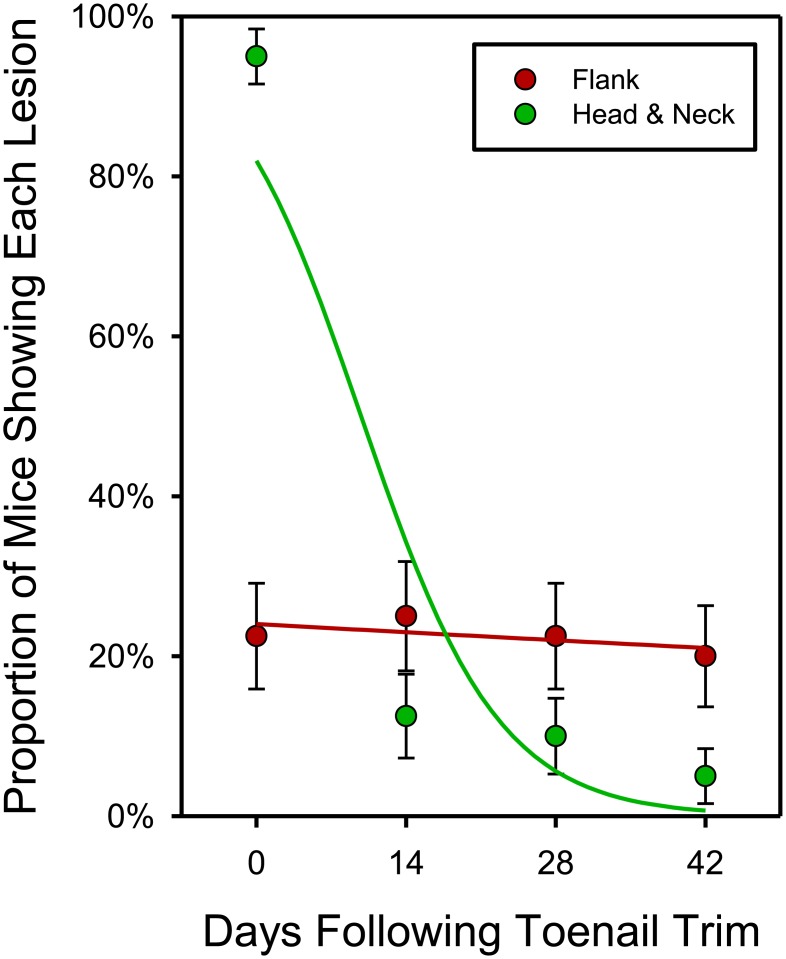
Resolution of lesion by type and as a function of time. Repeated-measures logistic regression showing the differential resolution of Flank versus Head & Neck lesions following Toenail Trim treatment. Each mouse was scored for each type of lesion every two weeks following first diagnosis. Although head-and-neck lesions were far more common at first diagnosis, they resolved rapidly, and did not recur (despite toenail regrowth). Flank lesions did not resolve (P = 0.0004). Observed Means and Standard Errors are plotted over the logistic regression curves.

Repeated measures logistic regression showed that the different lesion locations resolved differentially (Likelihood-Ratio ChiSquare = 24.43; P < 0.0001; Fig 4). Head-and-neck lesions improved rapidly over time (the odds of showing a head-and-neck lesion decreased cumulatively by approximately 14.3% a day: Likelihood-Ratio ChiSquare = 12.51; P = 0.0004). In contrast, flank lesions showed no evidence of resolving (the odds of showing a flank lesions decreased cumulatively by approximately 0.4% a day: Likelihood-Ratio ChiSquare = 0.46; P = 0.4962).

### Implementation Efficacy and the Impact of Topical Supplements

**Fig 5:**
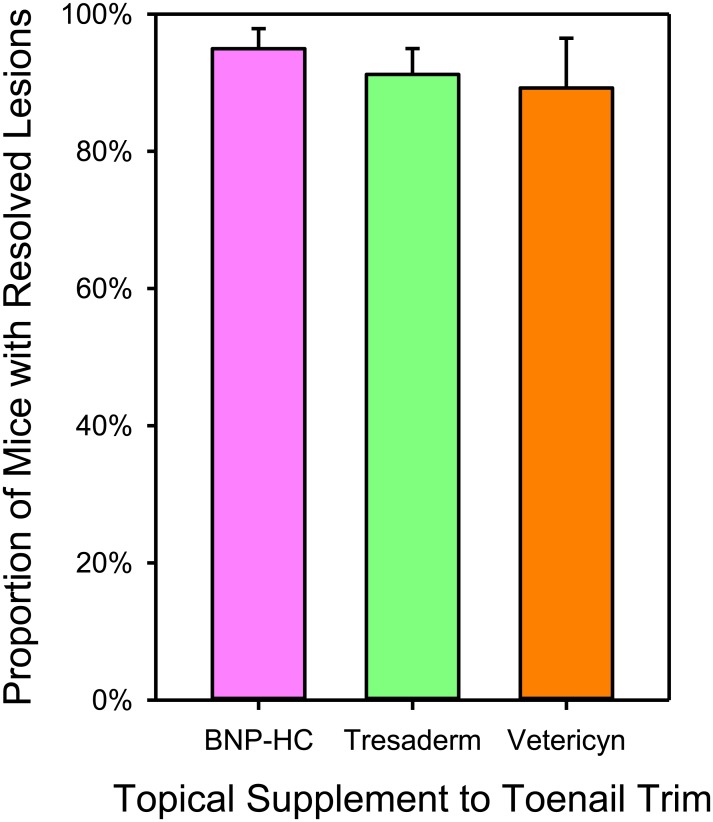
Lesion resolution as a function of topical supplement selected. Logistic regression analysis showing the proportion of all mice entering treatment that were cured rather than euthanized in the technician trial. All mice were toenail trimmed. However, depending on the veterinarian, a different topical was applied at the same time. Topical treatment had no effect on final cure rate (P = 0.6293). Estimated means and standard errors are shown from the logistic regression (which controls for date of first treatment, and duration of treatment).

**Fig 6:**
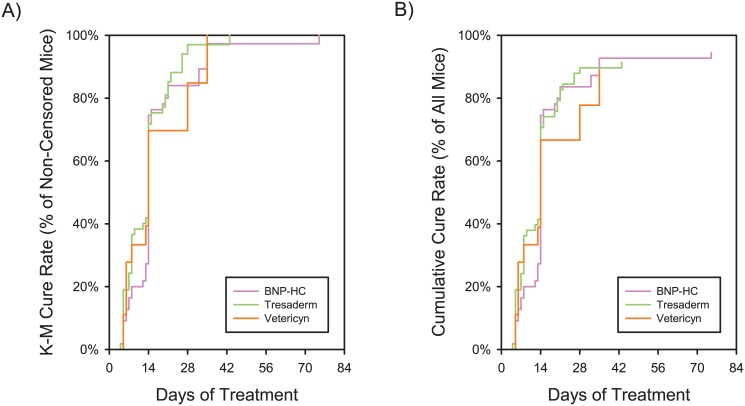
Lesion resolution as a function of time on treatment. A) Kaplan-Meier survivorship plot. Cure Rate is shown as the proportion of non-censored (i.e. non-euthanized) animals surviving at each time point. Euthanized animals are censored (i.e. removed from the total count at each time point). B) Cumulative Cure Rate, showing the raw cure events, without the artifacts injected by Kaplan-Meier survivorship calculations.

Logistic regression showed that cure rates did not differ significantly between mice receiving BNP-HC (95.0% +/- 2.9%), Tresaderm (91.2% +/- 3.8%) or Vetericyn (89.2% +/- 7.2%) (Likelihood-Ratio ChiSquare = 0.93; P < 0.6293; Fig 5). The Kaplan-Meier survivorship analysis found no difference in time to cure (all 3 topicals had a median time to cure of 14 days) (Log-Rank ChiSquare = 2.1282; P = 0.3450; Fig 6).

## Discussion

Toenail trims provide lasting and effective treatment that, when implemented properly, can be practical and efficient at an institutional level. Our findings have implications for the treatment of UD, a disease that has long frustrated scientists and veterinary staff alike owing to its recalcitrant responses to traditional wound therapies and poorly understood etiology. This treatment’s efficacy far exceeds that of our previous standard of care, daily topical Tresaderm, and indeed appears to exceed that of other treatments assessed in the literature. To our surprise, it was also largely curative, with no lesion recurrence observed during a prolonged observation period of 42 days during which the nails have regrown. Lastly, it can be performed effectively on an institutional level, with minimal time and effort, using any of the conventional topical supplements.

### Efficacy of Toenail Trims

Each of our aims built upon the central finding in this study that toenail trims are effective and result in lesion resolution of approximately 90% of treated mice. This level of efficacy comes as a surprise, especially in the face of this intervention’s simplicity. The UD literature often references toenail trims as a potential treatment option despite the fact that there is a paucity of data to support its efficacy. Poster presentations by Mufford et al. and Seta et al. provide the foundation on which this study rests; they reported toenail trims as a successful intervention strategy to combat UD at their facilities opening the way for further research in this area [19, 27]. Mufford et al. reported a decrease in time to heal and an increased number of animals responding to therapy relative to their previous standard of care [19]. However, the efficacy is not reported in the abstracts available for these studies so it is not possible to compare the toenail trim efficacy seen by this group and our own results.

Other treatments that have been employed as therapies for UD with reported efficacies include dietary vitamin E supplementation, ibuprofen, and maropitant citrate. Vitamin E supplementation resulted in 45% of 71 animals with complete re-epithelialization and hair regrowth at the lesion. The authors posited that vitamin E had a protective role in the oxidative damage that plays a role in the maintenance and progression of UD. The antioxidative effects of vitamin E interferes with the activation and production of potent inflammatory mediators and effectors including arachadonic acid, cyclooxygenase, and pro-inflammatory cytokines which may contribute to UD lesion appearance and maintenance. However, when this hypothesis was tested, UD lesions themselves did not differ in oxidative stress biomarkers from experimentally induced wounds [28–30]. Ibuprofen interferes with the cyclooxygenase-mediated elaboration of pro-inflammatory cytokines and therefore is thought to limit the inflammatory component of UD, which in turn inhibits some of the pruritic behavior associated with UD [15, 31]. Approximately 65% of the 15 animals receiving a liquid-gel formulation of Ibuprofen in water showed 50–100% lesion resolution by 9 days of treatment [15]. Maropitant citrate administration at 1mg/kg resulted in a reduction of pruritus and an increased odds of at least 25% improvement of lesion size relative to controls given water [20]. Substance P binding to NK1 receptors in mice has been shown to induce pruritic behaviors in mice [23]; maropitant citrate is an NK1 antagonist and disruption of this signaling was thought to decrease pruritus and therefore allow lesions to heal [20].

Each of the above strategies targets disruption of the itch-scratch cycle with varying degrees of success. Vitamin E, ibuprofen, and maropitant citrate appear to incompletely disrupt the cycle as evidenced by partial responses to therapy and incomplete cessation of pruritic behavior. Toenail trims, on the other hand, have approximately 90% efficacy and may have an impact on pruritus as well. We observed that mice that recover from lesions exhibit few signs of pruritus, which is the focus of our current follow-on study examining behavioral changes during lesion resolution. We believe that the toenail trim efficacy is due to a higher degree of blocking the itch-scratch cycle relative to other therapies. By removing the sharp, dagger-like toenail tips, mice are no longer capable of traumatizing dorsal neck lesions. Furthermore, by reducing the self-trauma attributable to the pruritic behavior, the itch-scratch cycle is completely halted. Without the constant trauma of scratching, we propose that pro-inflammatory signaling from self-wounding is gradually attenuated which results in decreased pruritic signaling at the lesion and unimpeded tissue healing. This has implications for the underlying cause of UD as we and others have hypothesized that behavior plays a dominant role in the pathology of UD [6, 8]. Furthermore, we have shown that both barbering and UD are associated with elevated oxidative stress in the brain, providing an explanation for the efficacy of antioxidants in the treatment of UD consistent with a behavioral mechanism for lesion onset and maintenance [32, 33].

The success of a mechanical intervention is significant support for the idea that UD lesions are intimately related to behavioral activity. While the triggering event that drives initial lesion formation remains elusive and is most likely multifactorial, we can be confident that the behavior of scratching is responsible for maintenance and progression of UD lesions as evidenced by the apparent reversal of lesions following nail trims. Furthermore, human patients with trichotillomania and Skin Picking Disorder show behavioral similarities to mouse barbering behavior and UD scratching behavior, respectively [6, 34].

### Toenail Trims and Recurrence

One of the more notable findings in this study is the lack of recurrence observed when animals were monitored every 14 days for 42 days following a single toenail trim. We hypothesized that lesions would recur following nail regrowth, requiring multiple nail trims for this treatment to be effective at controlling lesions. This hypothesis was formed on the basis of two premises. First, that toenails would regrow with time and, because we had not attempted to control any underlying pruritis, the mice would subsequently traumatize the skin again. Second, trimming the toenails is only palliative and underlying precipitative factors that may have tipped excessive scratching into self-wounding, would remain and cause lesion reappearance. Surprisingly, the lesions did not reappear following observable nail growth. This finding suggests that toenail trimming either a.) reduces the ongoing scratching behavior responsible for UD, b.) sufficiently interferes with the production of inflammatory mediators *in situ* such that pruritus is diminished and the scratching behavior is no longer elicited, or c.) plays some other, unknown role in behavior modulation that results in cessation of scratching behavior. These possibilities are not mutually exclusive. For instance, a combination of the first and second possibilities may be at play, where certain mice are predisposed to itching behavior, begin to itch due to some element of environment, genetics, or other factor, develop lesions that are maintained by the itch-scratch cycle, and by interrupting this process, toenail trimming halts the cycle. This interpretation is consistent with evidence that certain animals have a predisposition for scratching behavior that progresses to UD [6].

The recurrence study also revealed another interesting finding, the presence of flank lesions that do not resolve with toenail trim treatments. Flank lesions were always accompanied by dorsal neck lesions. However, they only appeared in approximately 20% of the animals. Furthermore, in these animals, the dorsal neck lesions resolved following treatment while the flank lesions worsened or stayed the same. In our records review for evaluating efficacy, it was frequently noted that the approximately 10% of animals that did not respond to therapy had flank lesions that were worsening and affecting ambulation. We also noted that during observation of these animals, they were frequently seen chewing at the fur and tissue of their flanks and thoracic limbs. This is an area of further interest for us as it represents a second mechanism of lesion progression and maintenance separate from scratching with pelvic limbs. We suspect that the pruritus associated with early neck lesions elicits scratching behaviors that nonspecifically traumatize nearby healthy tissue thereby expanding lesions. As they expand, lesions extend to areas that are beyond the reach of their toenails and in order to relieve the itch impulses, mice turn and chew at lesions. It may be that identifying typical dorsal neck lesions sooner and providing treatment early could prevent the progression of lesions to areas no longer amenable to treatment by toenail trims. In the final aim of our study, implementing toenail trimming at an institutional level by our technicians, we addressed this finding by considering UD flank lesions a grave prognosis and grounds for immediate euthanasia.

### Toenail Trims and Supplementation with Topical Treatments

When we began this study, we decided to include the use of a one-time topical treatment at the time of toenail trim because we believed it would provide at least temporary relief in addition to the long term relief provided by interrupting the itch-scratch cycle. At the conclusion of the records review, it became apparent that the addition of Vetericyn resulted in a confounding factor with regards to attributing outcome to the different steps of our treatment. In order to determine Vetericyn’s potential role in lesion resolution, we included BNP-HC and Tresaderm as alternative topical treatments to Vetericyn when implementing toenail trims at the institutional level. The results indicated that the choice of topical supplement had no impact on lesion resolution, and that all three resulted in approximately 90% of the mice with completely resolved UD lesions. We interpret this as toenail trims being the causal factor for lesion resolution rather than the supplemental topical.

### Institutional Implementation of Toenail Trims for UD Treatment

Once we observed that toenail trims were effective, we sought to make it practical. Initially, we used gas anesthetics provided by a rodent anesthesia machine to deliver the isoflurane to mice to provide chemical immobility for the toenail trims. This method presents several problems: availability of the anesthetic machines is limited owing to the high demand by our scientists, transportation of the anesthetic machine from facility to facility is a challenge due to the decentralized organization of our vivaria, and finally, anesthesia poses a risk, however small, for overdose and animal loss. As the pain and distress associated with the process of trimming toenails was thought to be mild and momentary, we believed the only advantage of anesthesia to be the chemical restraint of the animal for the fine motions required to trim. Towards that end, we set out to devise a restraint device that would enable users to immobilize the digits for toenail trimming without the use of anesthetics. During discussions with other veterinary services at other institutions attempting toenail trims, the limitations of restraint devices lie in the difficulty of restraining animals in a position that allows for quick trimming by a single person. 50 mL conical tubes have been attempted but found to be lacking as mice could rotate freely within and could still move their digits during trims even though their body was restrained within the tube. We addressed this by modifying the tube in such a manner as to engage the extensors of the distal pelvic limb when the knee is placed against the modified edge of the tube. As with several other animals (such as a dog), the biomechanics of the mouse knee is such that when it is straightened, the rest of the leg extends as well, including the toes. We used this to our advantage when designing the restraint device and found that trims could be performed quickly and reliably with a high degree of precision owing to the immobilization provided by the device.

Toenail trims have several other advantages that became apparent once we implemented their use at an institutional level. Treatments are cheap; the up-front cost of acquiring the tools necessary is much less than the cost of replenishing stocks of topicals that are exhausted rapidly with daily treatments. Furthermore, the man-hours spent performing toenail trims are minor compared to the time spent revisiting the same animals daily for topical treatments. We observed that, on average, an operator is able to perform the trims within 1 to 2 minutes from start to finish. Besides the cost savings, toenail trims also have the advantage of minimally interfering with research studies in contrast with systemic treatments, which may confound results. Additionally, performing a single treatment minimizes the stress of repeated handling and restraint for daily topical treatments.

## Limitations

Our examination of toenail trims as a treatment option for UD lesions had a few caveats. One important design limitation was the nature of the randomization of animals to the treatment groups. In this case we collected data following the implementation of new therapies in our animal care program, rather than during a prospective study with planned and blinded randomized treatment allocation. This is inherently the case in any records review study. Nevertheless, treatment allocation was essentially random owing to the rotation of veterinarians prescribing a particular treatment. By the same token, as these decisions were made by the veterinary staff in the past (i.e. prior to the records review), they were perfectly blinded as to the purpose of the experiment. Similarly, animal care staff monitoring the morbidity following treatment were aware of the treatment plan, but this was again before the records review so they were blinded to the expectations of the study. Finally, as our Standard Operating Procedures specify objectively measurable humane endpoints for UD, the impact of knowing the treatment plan on the decision to euthanize will have been minimal.

Furthermore, we could not analyze lesion severity in our records reviews. This was a due to the data available from our written medical records, which gave diagnosis, but not severity. However, given the large number of animals that responded to therapy between our three experiments, we can safely assume that a wide spectrum of clinical severities is included in this study.

Another caveat is the lack of a placebo control group to compare our treatments against. We believed that administering a placebo treatment was unethical in mice with UD and therefore chose a current-standard-of-care control (Tresaderm) instead. A related limitation was the lack of a nail trim alone treatment group as we provided a single application of topical at the time of trimming. We opted for a one time topical application to provide multimodal treatment with the nail trim at the time of care, because this is the ethical real-world approach to these cases; and we wanted to test whether toenail trimming was a viable real-world treatment strategy.

A further limitation of the study was our comparison with Tresaderm given daily rather than Vetericyn in the first records review. At the time, Tresaderm was our standard of care and there were a large number of medical records to compare outcome to; on the other hand, Vetericyn was only introduced as a treatment option to our facilities when we first started performing toenail trims. Most of our veterinarians had a preference for prescribing Tresaderm versus the untried Vetericyn. However, the third experiment which compared supplemental topicals allowed us to rule out the particular topical used as a confounding variable, and conclude that the effect of lesion resolution was due to the toenail trim treatment itself.

## Conclusions

Our study has provided solid evidence for the use of toenail trims as a treatment option for UD lesions that results in complete lesion resolution in approximately 90% of the mice treated. Furthermore, the lesions do not recur with time. While flank lesions appear refractory to treatment, identification of these lesions justifies the provision of early euthanasia rather than prolonged, ineffective treatment. Treatments can be performed efficiently and practically with the use of a restraint device that allows a single person to provide the one time toenail trim. Finally, the success of a mechanical intervention provides strong support for the maintenance and progression of UD lesions as having a behavioral origin.

## Supporting Information

S1 Dataset Dataset analyzed in this study. (DOCX)

S1 Video Video demonstrating toenail trim procedure and use of the mouse restraint device. (MP4)
