# Supplementary material for: A “Pedi” Cures All: Toenail Trimming and the Treatment of Ulcerative Dermatitis in Mice
Source: PLoS One. 2016 Jan 6;11(1):e0144871. doi: 10.1371/journal.pone.0144871 (PMC4703297; doi:10.1371/journal.pone.0144871)
Supplement: S1 Dataset — (DOCX) [file pone.0144871.s001.docx]

# Supplementary data

The raw data for each of the analyses are presented below. Each data set is given as a SAS code for the data and analysis itself. Data are presented in SAS format as this is a simple text format.

# Experiment 1: Records Review

**DATA** RecordReview_1_year_20141208; INPUT Tx &$16. First_Treatment DaysToOutcome Outcome &$16. Censor; Lines;

TNT **3490732800** **34** Cure **1**

TNT **3491942400** **49** Cure **1**

Tresaderm **3495139200** **14** Euthanasia **0**

TNT **3492720000** **20** Cure **1**

TNT **3492720000** **20** Cure **1**

Tresaderm **3487708800** **6** Cure **1**

Tresaderm **3490560000** **7** Cure **1**

Tresaderm **3476304000** **19** Cure **1**

Tresaderm **3476995200** **14** Cure **1**

Tresaderm **3480537600** **13** Euthanasia **0**

Tresaderm **3480969600** **14** Euthanasia **0**

Tresaderm **3480537600** **18** Euthanasia **0**

Tresaderm **3479328000** **21** Euthanasia **0**

TNT **3478118400** **27** Cure **1**

TNT **3479068800** **31** Cure **1**

TNT **3489609600** **13** Cure **1**

TNT **3490214400** **22** Cure **1**

TNT **3486672000** **20** Cure **1**

TNT **3484252800** **13** Cure **1**

TNT **3484512000** **14** Cure **1**

TNT **3484252800** **13** Cure **1**

TNT **3478550400** **43** Euthanasia **0**

Tresaderm **3479846400** **7** Euthanasia **0**

Tresaderm **3477254400** **3** Euthanasia **0**

Tresaderm **3493756800** **2** Euthanasia **0**

Tresaderm **3479328000** **13** Euthanasia **0**

Tresaderm **3482265600** **7** Euthanasia **0**

TNT **3489523200** **14** Cure **1**

TNT **3495571200** **16** Cure **1**

TNT **3495398400** **11** Cure **1**

TNT **3495398400** **11** Cure **1**

Tresaderm **3496780800** **5** Euthanasia **0**

TNT **3497558400** **14** Cure **1**

Tresaderm **3476217600** **5** Euthanasia **0**

Tresaderm **3496089600** **2** Euthanasia **0**

TNT **3497299200** **14** Cure **1**

TNT **3497385600** **12** Cure **1**

TNT **3495052800** **15** Cure **1**

TNT **3495052800** **28** Cure **1**

TNT **3497472000** **14** Cure **1**

Tresaderm **3493238400** **8** Euthanasia **0**

TNT **3490560000** **11** Cure **1**

TNT **3486067200** **25** Cure **1**

Tresaderm **3487276800** **6** Euthanasia **0**

TNT **3482438400** **13** Cure **1**

TNT **3480883200** **16** Euthanasia **0**

TNT **3478723200** **18** Cure **1**

TNT **3478723200** **18** Cure **1**

TNT **3478723200** **18** Cure **1**

TNT **3476304000** **28** Cure **1**

TNT **3476304000** **28** Cure **1**

TNT **3476995200** **20** Cure **1**

TNT **3476304000** **15** Cure **1**

Tresaderm **3474576000** **14** Euthanasia **0**

Tresaderm **3471552000** **14** Euthanasia **0**

TNT **3469132800** **11** Cure **1**

Tresaderm **3484080000** **6** Euthanasia **0**

Tresaderm **3482179200** **10** Euthanasia **0**

Tresaderm **3480451200** **6** Euthanasia **0**

Tresaderm **3480451200** **7** Euthanasia **0**

Tresaderm **3496608000** **14** Euthanasia **0**

TNT **3484598400** **15** Euthanasia **0**

TNT **3485980800** **12** Cure **1**

Tresaderm **3486326400** **15** Cure **1**

Tresaderm **3496780800** **5** Euthanasia **0**

Tresaderm **3470169600** **19** Euthanasia **0**

Tresaderm **3476131200** **27** Euthanasia **0**

Tresaderm **3485894400** **8** Cure **1**

Tresaderm **3479932800** **11** Cure **1**

Tresaderm **3468268800** **13** Euthanasia **0**

Tresaderm **3470083200** **10** Cure **1**

Tresaderm **3473366400** **12** Euthanasia **0**

Tresaderm **3487881600** **11** Euthanasia **0**

TNT **3495052800** **15** Cure **1**

Tresaderm **3478464000** **9** Cure **1**

Tresaderm **3482265600** **14** Cure **1**

Tresaderm **3492115200** **7** Cure **1**

Tresaderm **3470342400** **25** Cure **1**

Tresaderm **3473280000** **8** Cure **1**

Tresaderm **3493756800** **14** Euthanasia **0**

Tresaderm **3474403200** **22** Cure **1**

Tresaderm **3481142400** **25** Euthanasia **0**

Tresaderm **3482438400** **53** Euthanasia **0**

Tresaderm **3469046400** **13** Euthanasia **0**

Tresaderm **3471292800** **14** Euthanasia **0**

Tresaderm **3471292800** **14** Euthanasia **0**

Tresaderm **3471552000** **13** Euthanasia **0**

Tresaderm **3473193600** **21** Euthanasia **0**

Tresaderm **3475094400** **8** Euthanasia **0**

Tresaderm **3473798400** **26** Euthanasia **0**

Tresaderm **3473884800** **29** Euthanasia **0**

Tresaderm **3475699200** **7** Euthanasia **0**

Tresaderm **3478032000** **5** Euthanasia **0**

Tresaderm **3479241600** **12** Euthanasia **0**

Tresaderm **3479241600** **13** Euthanasia **0**

Tresaderm **3478636800** **27** Euthanasia **0**

Tresaderm **3479760000** **14** Euthanasia **0**

Tresaderm **3480364800** **14** Euthanasia **0**

Tresaderm **3472588800** **14** Cure **1**

TNT **3497904000** **14** Cure **1**

Tresaderm **3482438400** **26** Cure **1**

Tresaderm **3471552000** **12** Cure **1**

Tresaderm **3482956800** **6** Cure **1**

Tresaderm **3487881600** **12** Euthanasia **0**

Tresaderm **3468873600** **15** Euthanasia **0**

Tresaderm **3470601600** **16** Euthanasia **0**

Tresaderm **3470860800** **15** Euthanasia **0**

Tresaderm **3471811200** **16** Euthanasia **0**

Tresaderm **3486931200** **14** Euthanasia **0**

Tresaderm **3490560000** **9** Euthanasia **0**

Tresaderm **3469564800** **16** Euthanasia **0**

Tresaderm **3471292800** **8** Euthanasia **0**

Tresaderm **3472675200** **29** Euthanasia **0**

Tresaderm **3474230400** **22** Cure **1**

Tresaderm **3475440000** **25** Euthanasia **0**

Tresaderm **3475440000** **32** Euthanasia **0**

Tresaderm **3482265600** **5** Cure **1**

Tresaderm **3484166400** **13** Euthanasia **0**

Tresaderm **3484166400** **13** Euthanasia **0**

Tresaderm **3488400000** **14** Euthanasia **0**

Tresaderm **3488400000** **14** Euthanasia **0**

Tresaderm **3488400000** **8** Cure **1**

Tresaderm **3488400000** **13** Euthanasia **0**

Tresaderm **3489091200** **24** Euthanasia **0**

Tresaderm **3491164800** **21** Cure **1**

Tresaderm **3496867200** **14** Euthanasia **0**

Tresaderm **3475699200** **5** Cure **1**

Tresaderm **3481747200** **6** Cure **1**

Tresaderm **3484080000** **12** Euthanasia **0**

Tresaderm **3472156800** **19** Euthanasia **0**

Tresaderm **3478118400** **4** Euthanasia **0**

Tresaderm **3479673600** **7** Cure **1**

Tresaderm **3470601600** **17** Euthanasia **0**

Tresaderm **3468960000** **9** Euthanasia **0**

Tresaderm **3468182400** **18** Euthanasia **0**

Tresaderm **3472156800** **17** Euthanasia **0**

Tresaderm **3490732800** **14** Cure **1**

;

**RUN**;

**PROC** **GENMOD** DATA=RecordReview_1_year_20141208;

CLASS Tx;

MODEL Outcome = Tx First_Treatment DaysToOutcome/ DIST=Binomial LINK=Logit type3;

lsmeans tx / diff ilink oddsratio;

**RUN**;

**PROC** **lifetest** DATA=RecordReview_1_year_20141208;

Time daystooutcome*censor(**1**);

Strata TX;

**RUN**;

# Experiment 2: UD Recurrence, Lesion Type, and Treatment Efficacy

**DATA** Repeated_measures_outcome_6_wee; INPUT Barcode &$9. Day LesionType &$16. LesionSatus &$16.; Lines;

**3347935** **0** Flank Resolved

**3347935** **0** Head Present

**3347935** **14** Flank Resolved

**3347935** **14** Head Resolved

**3347935** **28** Flank Resolved

**3347935** **28** Head Resolved

**3347935** **42** Flank Resolved

**3347935** **42** Head Resolved

**3499643** **0** Flank Present

**3499643** **0** Head Present

**3499643** **14** Flank Present

**3499643** **14** Head Present

**3499643** **28** Flank Present

**3499643** **28** Head Present

**3499643** **42** Flank Present

**3499643** **42** Head Present

**3524561**-**1** **0** Flank Resolved

**3524561**-**1** **0** Head Present

**3524561**-**1** **14** Flank Present

**3524561**-**1** **14** Head Present

**3524561**-**1** **28** Flank Present

**3524561**-**1** **28** Head Present

**3524561**-**1** **42** Flank Present

**3524561**-**1** **42** Head Resolved

**3524561**-**2** **0** Flank Present

**3524561**-**2** **0** Head Present

**3524561**-**2** **14** Flank Present

**3524561**-**2** **14** Head Present

**3524561**-**2** **28** Flank Present

**3524561**-**2** **28** Head Present

**3524561**-**2** **42** Flank Present

**3524561**-**2** **42** Head Resolved

**3524561**-**3** **0** Flank Resolved

**3524561**-**3** **0** Head Present

**3524561**-**3** **14** Flank Resolved

**3524561**-**3** **14** Head Resolved

**3524561**-**3** **28** Flank Resolved

**3524561**-**3** **28** Head Resolved

**3524561**-**3** **42** Flank Resolved

**3524561**-**3** **42** Head Resolved

**3524564** **0** Flank Resolved

**3524564** **0** Head Present

**3524564** **14** Flank Resolved

**3524564** **14** Head Resolved

**3524564** **28** Flank Resolved

**3524564** **28** Head Resolved

**3524564** **42** Flank Resolved

**3524564** **42** Head Resolved

**3524567**-**1** **0** Flank Resolved

**3524567**-**1** **0** Head Present

**3524567**-**1** **14** Flank Resolved

**3524567**-**1** **14** Head Resolved

**3524567**-**1** **28** Flank Resolved

**3524567**-**1** **28** Head Resolved

**3524567**-**1** **42** Flank Resolved

**3524567**-**1** **42** Head Resolved

**3524567**-**2** **0** Flank Present

**3524567**-**2** **0** Head Present

**3524567**-**2** **14** Flank Present

**3524567**-**2** **14** Head Resolved

**3524567**-**2** **28** Flank Present

**3524567**-**2** **28** Head Resolved

**3524567**-**2** **42** Flank Present

**3524567**-**2** **42** Head Resolved

**3524568**-**1** **0** Flank Present

**3524568**-**1** **0** Head Present

**3524568**-**1** **14** Flank Present

**3524568**-**1** **14** Head Present

**3524568**-**1** **28** Flank Present

**3524568**-**1** **28** Head Present

**3524568**-**1** **42** Flank Present

**3524568**-**1** **42** Head Present

**3524568**-**2** **0** Flank Resolved

**3524568**-**2** **0** Head Present

**3524568**-**2** **14** Flank Resolved

**3524568**-**2** **14** Head Resolved

**3524568**-**2** **28** Flank Resolved

**3524568**-**2** **28** Head Resolved

**3524568**-**2** **42** Flank Resolved

**3524568**-**2** **42** Head Resolved

**3525707** **0** Flank Present

**3525707** **0** Head Present

**3525707** **14** Flank Present

**3525707** **14** Head Resolved

**3525707** **28** Flank Present

**3525707** **28** Head Resolved

**3525707** **42** Flank Present

**3525707** **42** Head Resolved

**3538701** **0** Flank Resolved

**3538701** **0** Head Present

**3538701** **14** Flank Resolved

**3538701** **14** Head Resolved

**3538701** **28** Flank Resolved

**3538701** **28** Head Resolved

**3538701** **42** Flank Resolved

**3538701** **42** Head Resolved

**3538703** **0** Flank Resolved

**3538703** **0** Head Present

**3538703** **14** Flank Resolved

**3538703** **14** Head Resolved

**3538703** **28** Flank Resolved

**3538703** **28** Head Resolved

**3538703** **42** Flank Resolved

**3538703** **42** Head Resolved

**3541660** **0** Flank Resolved

**3541660** **0** Head Present

**3541660** **14** Flank Present

**3541660** **14** Head Present

**3541660** **28** Flank Present

**3541660** **28** Head Resolved

**3541660** **42** Flank Resolved

**3541660** **42** Head Resolved

**3541661** **0** Flank Resolved

**3541661** **0** Head Present

**3541661** **14** Flank Resolved

**3541661** **14** Head Resolved

**3541661** **28** Flank Resolved

**3541661** **28** Head Resolved

**3541661** **42** Flank Resolved

**3541661** **42** Head Resolved

**3541662** **0** Flank Present

**3541662** **0** Head Present

**3541662** **14** Flank Resolved

**3541662** **14** Head Resolved

**3541662** **28** Flank Resolved

**3541662** **28** Head Resolved

**3541662** **42** Flank Resolved

**3541662** **42** Head Resolved

**3541665** **0** Flank Resolved

**3541665** **0** Head Present

**3541665** **14** Flank Resolved

**3541665** **14** Head Resolved

**3541665** **28** Flank Resolved

**3541665** **28** Head Resolved

**3541665** **42** Flank Resolved

**3541665** **42** Head Resolved

**3541667** **0** Flank Present

**3541667** **0** Head Present

**3541667** **14** Flank Present

**3541667** **14** Head Resolved

**3541667** **28** Flank Present

**3541667** **28** Head Resolved

**3541667** **42** Flank Present

**3541667** **42** Head Resolved

**3541669**-**1** **0** Flank Resolved

**3541669**-**1** **0** Head Present

**3541669**-**1** **14** Flank Resolved

**3541669**-**1** **14** Head Resolved

**3541669**-**1** **28** Flank Resolved

**3541669**-**1** **28** Head Resolved

**3541669**-**1** **42** Flank Resolved

**3541669**-**1** **42** Head Resolved

**3541669**-**2** **0** Flank Resolved

**3541669**-**2** **0** Head Present

**3541669**-**2** **14** Flank Resolved

**3541669**-**2** **14** Head Resolved

**3541669**-**2** **28** Flank Resolved

**3541669**-**2** **28** Head Resolved

**3541669**-**2** **42** Flank Resolved

**3541669**-**2** **42** Head Resolved

**3541670**-**1** **0** Flank Resolved

**3541670**-**1** **0** Head Present

**3541670**-**1** **14** Flank Resolved

**3541670**-**1** **14** Head Resolved

**3541670**-**1** **28** Flank Resolved

**3541670**-**1** **28** Head Resolved

**3541670**-**1** **42** Flank Resolved

**3541670**-**1** **42** Head Resolved

**3541670**-**2** **0** Flank Resolved

**3541670**-**2** **0** Head Present

**3541670**-**2** **14** Flank Resolved

**3541670**-**2** **14** Head Resolved

**3541670**-**2** **28** Flank Resolved

**3541670**-**2** **28** Head Resolved

**3541670**-**2** **42** Flank Resolved

**3541670**-**2** **42** Head Resolved

**3541671** **0** Flank Present

**3541671** **0** Head Present

**3541671** **14** Flank Present

**3541671** **14** Head Resolved

**3541671** **28** Flank Resolved

**3541671** **28** Head Resolved

**3541671** **42** Flank Resolved

**3541671** **42** Head Resolved

**3541673**-**1** **0** Flank Resolved

**3541673**-**1** **0** Head Present

**3541673**-**1** **14** Flank Resolved

**3541673**-**1** **14** Head Resolved

**3541673**-**1** **28** Flank Resolved

**3541673**-**1** **28** Head Resolved

**3541673**-**1** **42** Flank Resolved

**3541673**-**1** **42** Head Resolved

**3541673**-**2** **0** Flank Resolved

**3541673**-**2** **0** Head Present

**3541673**-**2** **14** Flank Resolved

**3541673**-**2** **14** Head Resolved

**3541673**-**2** **28** Flank Resolved

**3541673**-**2** **28** Head Resolved

**3541673**-**2** **42** Flank Resolved

**3541673**-**2** **42** Head Resolved

**3541674**-**1** **0** Flank Resolved

**3541674**-**1** **0** Head Resolved

**3541674**-**1** **14** Flank Resolved

**3541674**-**1** **14** Head Resolved

**3541674**-**1** **28** Flank Resolved

**3541674**-**1** **28** Head Resolved

**3541674**-**1** **42** Flank Resolved

**3541674**-**1** **42** Head Resolved

**3541674**-**2** **0** Flank Resolved

**3541674**-**2** **0** Head Resolved

**3541674**-**2** **14** Flank Resolved

**3541674**-**2** **14** Head Resolved

**3541674**-**2** **28** Flank Resolved

**3541674**-**2** **28** Head Resolved

**3541674**-**2** **42** Flank Resolved

**3541674**-**2** **42** Head Resolved

**3541677**-**1** **0** Flank Resolved

**3541677**-**1** **0** Head Present

**3541677**-**1** **14** Flank Resolved

**3541677**-**1** **14** Head Resolved

**3541677**-**1** **28** Flank Resolved

**3541677**-**1** **28** Head Resolved

**3541677**-**1** **42** Flank Resolved

**3541677**-**1** **42** Head Resolved

**3541677**-**2** **0** Flank Resolved

**3541677**-**2** **0** Head Present

**3541677**-**2** **14** Flank Resolved

**3541677**-**2** **14** Head Resolved

**3541677**-**2** **28** Flank Resolved

**3541677**-**2** **28** Head Resolved

**3541677**-**2** **42** Flank Resolved

**3541677**-**2** **42** Head Resolved

**3541677**-**3** **0** Flank Resolved

**3541677**-**3** **0** Head Present

**3541677**-**3** **14** Flank Resolved

**3541677**-**3** **14** Head Resolved

**3541677**-**3** **28** Flank Resolved

**3541677**-**3** **28** Head Resolved

**3541677**-**3** **42** Flank Resolved

**3541677**-**3** **42** Head Resolved

**3543543** **0** Flank Resolved

**3543543** **0** Head Present

**3543543** **14** Flank Resolved

**3543543** **14** Head Resolved

**3543543** **28** Flank Resolved

**3543543** **28** Head Resolved

**3543543** **42** Flank Resolved

**3543543** **42** Head Resolved

**3554062** **0** Flank Resolved

**3554062** **0** Head Present

**3554062** **14** Flank Resolved

**3554062** **14** Head Resolved

**3554062** **28** Flank Resolved

**3554062** **28** Head Resolved

**3554062** **42** Flank Resolved

**3554062** **42** Head Resolved

**3554063** **0** Flank Resolved

**3554063** **0** Head Present

**3554063** **14** Flank Resolved

**3554063** **14** Head Resolved

**3554063** **28** Flank Resolved

**3554063** **28** Head Resolved

**3554063** **42** Flank Resolved

**3554063** **42** Head Resolved

**3554069** **0** Flank Resolved

**3554069** **0** Head Present

**3554069** **14** Flank Resolved

**3554069** **14** Head Resolved

**3554069** **28** Flank Resolved

**3554069** **28** Head Resolved

**3554069** **42** Flank Resolved

**3554069** **42** Head Resolved

**3554070** **0** Flank Resolved

**3554070** **0** Head Present

**3554070** **14** Flank Resolved

**3554070** **14** Head Resolved

**3554070** **28** Flank Resolved

**3554070** **28** Head Resolved

**3554070** **42** Flank Resolved

**3554070** **42** Head Resolved

**3554072** **0** Flank Resolved

**3554072** **0** Head Present

**3554072** **14** Flank Resolved

**3554072** **14** Head Resolved

**3554072** **28** Flank Resolved

**3554072** **28** Head Resolved

**3554072** **42** Flank Resolved

**3554072** **42** Head Resolved

**3554074**-**1** **0** Flank Resolved

**3554074**-**1** **0** Head Present

**3554074**-**1** **14** Flank Resolved

**3554074**-**1** **14** Head Resolved

**3554074**-**1** **28** Flank Resolved

**3554074**-**1** **28** Head Resolved

**3554074**-**1** **42** Flank Resolved

**3554074**-**1** **42** Head Resolved

**3554074**-**2** **0** Flank Resolved

**3554074**-**2** **0** Head Present

**3554074**-**2** **14** Flank Resolved

**3554074**-**2** **14** Head Resolved

**3554074**-**2** **28** Flank Resolved

**3554074**-**2** **28** Head Resolved

**3554074**-**2** **42** Flank Resolved

**3554074**-**2** **42** Head Resolved

**3554075** **0** Flank Present

**3554075** **0** Head Present

**3554075** **14** Flank Present

**3554075** **14** Head Resolved

**3554075** **28** Flank Present

**3554075** **28** Head Resolved

**3554075** **42** Flank Present

**3554075** **42** Head Resolved

**3559945** **0** Flank Resolved

**3559945** **0** Head Present

**3559945** **14** Flank Resolved

**3559945** **14** Head Resolved

**3559945** **28** Flank Resolved

**3559945** **28** Head Resolved

**3559945** **42** Flank Resolved

**3559945** **42** Head Resolved

;

**RUN**;

**PROC** **GENMOD** DATA=Repeated_measures_outcome_6_wee;

CLASS Barcode LesionType;

MODEL LesionSatus = Day LesionType Day*LesionType/ DIST=Binomial LINK=Logit TYpe3;

repeated subject=barcode;

lsmeans lesiontype / at day=**0** ilink plot=none;

lsmeans lesiontype / at day=**14** ilink plot=none;

lsmeans lesiontype / at day=**28** ilink plot=none;

lsmeans lesiontype / at day=**42** ilink plot=none;

lsmeans lesiontype / at day=**56** ilink plot=none;

lsmeans lesiontype / at day=**70** ilink plot=none;

estimate 'Head slope' day **1** day*lesiontype -**0** **1**/ e;

estimate 'Flank slope' day **1** day*lesiontype **1** **0**/ e;

estimate 'mean slope' day **1** / e;

**RUN**;

# Experiment 3: Implementation Efficacy and the Impact of Topical Supplements

**DATA** Technician_trial_20150226; INPUT Date Topical_used &$16. Outcome &$16. TimeToEvent Censored; Lines;

**3533068800** T Cured **43** **0**

**3503865600** B Cured **8** **0**

**3498681600** B Cured **4** **0**

**3504211200** B Cured **75** **0**

**3499286400** T Cured **14** **0**

**3499200000** T Cured **14** **0**

**3499286400** T Cured **14** **0**

**3499286400** T Cured **14** **0**

**3499286400** T Cured **14** **0**

**3499286400** T Cured **14** **0**

**3499286400** T Cured **14** **0**

**3499286400** T Cured **14** **0**

**3499286400** T Cured **14** **0**

**3497817600** T Euthansia **20** **1**

**3497817600** T Cured **20** **0**

**3497817600** T Cured **20** **0**

**3500496000** B Cured **14** **0**

**3500496000** B Cured **14** **0**

**3500496000** B Cured **14** **0**

**3501705600** B Cured **14** **0**

**3501705600** B Cured **14** **0**

**3501705600** B Cured **14** **0**

**3501705600** B Cured **14** **0**

**3501705600** B Cured **14** **0**

**3501705600** B Cured **14** **0**

**3501705600** B Cured **14** **0**

**3501705600** B Cured **14** **0**

**3501705600** B Cured **14** **0**

**3501705600** B Cured **14** **0**

**3501705600** B Cured **14** **0**

**3501705600** B Cured **14** **0**

**3501705600** B Cured **14** **0**

**3502310400** B Cured **14** **0**

**3502310400** B Cured **14** **0**

**3502310400** B Cured **14** **0**

**3502310400** B Cured **14** **0**

**3502310400** B Cured **14** **0**

**3502310400** B Cured **14** **0**

**3502310400** B Cured **14** **0**

**3503952000** T Cured **5** **0**

**3503952000** T Cured **5** **0**

**3503952000** T Cured **5** **0**

**3501705600** B Cured **32** **0**

**3503433600** B Cured **5** **0**

**3499286400** T Cured **14** **0**

**3499286400** T Cured **14** **0**

**3499113600** T Cured **8** **0**

**3498076800** T Cured **21** **0**

**3498076800** T Cured **21** **0**

**3498076800** B Cured **21** **0**

**3498076800** B Cured **21** **0**

**3498076800** T Cured **8** **0**

**3498076800** T Cured **8** **0**

**3498076800** T Cured **8** **0**

**3498076800** T Cured **8** **0**

**3498076800** T Cured **8** **0**

**3500496000** B Cured **20** **0**

**3499804800** T Euthansia **21** **1**

**3499804800** T Euthansia **21** **1**

**3499027200** T Cured **28** **0**

**3502828800** T Cured **7** **0**

**3499286400** T Cured **19** **0**

**3498940800** V Cured **28** **0**

**3504124800** V Euthansia **14** **1**

**3504729600** B Cured **5** **0**

**3503520000** V Cured **28** **0**

**3503520000** V Cured **14** **0**

**3501014400** T Cured **5** **0**

**3498681600** B Cured **12** **0**

**3502224000** T Euthansia **15** **1**

**3502828800** V Cured **14** **0**

**3502224000** T Cured **14** **0**

**3499718400** V Cured **14** **0**

**3498422400** B Cured **6** **0**

**3502656000** T Cured **5** **0**

**3501014400** B Cured **15** **0**

**3500582400** B Cured **5** **0**

**3500236800** V Cured **8** **0**

**3500409600** B Cured **6** **0**

**3504729600** V Cured **13** **0**

**3500236800** T Euthansia **6** **1**

**3498681600** T Cured **26** **0**

**3500323200** T Cured **7** **0**

**3500236800** T Cured **8** **0**

**3497817600** V Cured **6** **0**

**3497817600** V Cured **6** **0**

**3497817600** V Cured **6** **0**

**3500323200** T Cured **12** **0**

**3501014400** T Cured **5** **0**

**3501014400** T Cured **5** **0**

**3502051200** B Cured **7** **0**

**3500928000** T Cured **26** **0**

**3502137600** B Cured **13** **0**

**3502137600** B Cured **13** **0**

**3503865600** B Cured **8** **0**

**3499632000** T Cured **14** **0**

**3499632000** T Cured **15** **0**

**3504556800** V Cured **5** **0**

**3504556800** V Cured **5** **0**

**3499027200** B Cured **35** **0**

**3499027200** B Cured **35** **0**

**3499027200** B Cured **35** **0**

**3502656000** B Cured **14** **0**

**3498508800** T Cured **7** **0**

**3499804800** V Cured **35** **0**

**3499804800** V Cured **35** **0**

**3502224000** T Cured **15** **0**

**3503433600** B Cured **5** **0**

**3501014400** B Euthansia **20** **1**

**3501014400** B Cured **19** **0**

**3499804800** V Cured **14** **0**

**3503001600** B Euthansia **21** **1**

**3503001600** B Euthansia **21** **1**

**3502051200** B Cured **7** **0**

**3499459200** T Cured **22** **0**

**3499459200** T Cured **14** **0**

**3503347200** B Cured **14** **0**

**3499200000** T Cured **14** **0**

**3505161600** V Euthansia **10** **1**

**3499113600** T Cured **5** **0**

**3503952000** T Cured **13** **0**

**3503952000** B Cured **13** **0**

**3499286400** V Cured **14** **0**

**3501619200** T Cured **4** **0**

**3499113600** T Cured **14** **0**

**3498854400** T Cured **14** **0**

**3498508800** T Cured **9** **0**

**3499200000** T Cured **5** **0**

**3499200000** T Cured **5** **0**

**3499977600** B Cured **14** **0**

**3499200000** B Cured **32** **0**

;

**RUN**;

**PROC** **GENMOD** DATA=Technician_trial_20150226;

CLASS Topical_used;

MODEL Outcome = Date TimeToEvent Topical_used/ DIST=Binomial LINK=Logit type3;

lsmeans Topical_used / ilink;

**RUN**;

**PROC** **lifetest** DATA=Technician_trial_20150226;

Time TimeToEvent*censored(**1**);

Strata Topical_used;

**RUN**;
